# Promoting Self-Determination in Young Adults with Autism: A Multicenter, Mixed Methods Study

**DOI:** 10.1007/s10803-025-06739-6

**Published:** 2025-02-15

**Authors:** Clara Andrés-Gárriz, Núria Farriols Hernando, Antonia Maria Gómez Hinojosa, Teresa Pretel-Luque, Sergi Fàbregues, Cristina Mumbardó-Adam

**Affiliations:** 1 Faculty of Psychology, Sciences of Education and Sports, Blanquerna, Universitat Ramon Llull, 34 Císter Street, Barcelona, Catalonia, 08022 Spain; 2 Department of Cognition, Development and Education Psychology, Faculty of Psychology, University of Barcelona, Barcelona, Catalonia, Spain; 3 Hospital of Mataró, Consorci Sanitari del Maresme, Mataró, Catalonia, Spain; 4 Department of Psychology and Education, Universitat Oberta de Catalunya (UOC), Barcelona, Catalonia, Spain

**Keywords:** Autism, Self-determination, Supports, Intervention, Mixed methods

## Abstract

Young people with autism have support needs related to self-determination that are currently not adequately addressed in Spain. This study aimed to assess the effectiveness, implementation, and acceptability of a program to support self-determination for young adults with autism in Spain. A multicenter, mixed methods randomized controlled trial (RCT) study was conducted (2020–2022). Young adults with autism between 17 and 30 years of age were recruited, and 40 were randomly assigned to the intervention or waiting-list group. Quantitative and qualitative data on outcomes relating to self-determination, program implementation and acceptability were collected at baseline, during, and after the intervention using several tools, including the SDI: SR and focus groups. Joint displays were used to integrate the quantitative and qualitative results for a comprehensive evaluation of the program. The quantitative results revealed no significant differences between the intervention and waiting list groups but indicated positive impacts on agentic actions and overall self-determination as reported by participants and caregivers. The qualitative results expanded the quantitative results by identifying personal and contextual barriers and facilitators of self-determination while offering deeper insights into the quantitative outcomes. Implementation fidelity was high, and the qualitative data provided areas for improvement and identified challenges and best practices. Program acceptability was high, and the group format proved useful. Our study provides the first empirical evidence of an intervention designed to promote self-determination in individuals with autism in Spain, but further research is needed. This trial was retrospectively registered at www.clinicaltrial.org (NCT05938751) on January 1^st^, 2023.

**Supplementary Information:**

The online version contains supplementary material available at 10.1007/s10803-025-06739-6.

Young adults with autism face multiple challenges during their transition to adulthood which significantly impact their quality of life, such as finding employment, living independently, and accessing necessary support, (QoL; Curtiss et al., 2021; Lachapelle et al., 2005; Lee et al., 2023; Mason et al., 2018; Wei et al., 2015). Self-determination is a key personal trait that is strongly related to enhanced QoL and can help people with autism navigate this transition (Shogren & Shaw, 2016; Shogren et al., 2015b). The literature has shown that self-determination contributes to QoL enhancement when it is positively promoted, for example, by supporting the development of personal talent and skills related to self-determination and causal agency (Mumbardó-Adam et al., 2024).

According to Causal Agency Theory (CAT), a framework that aims to understand and promote self-determined behavior on individuals with and without disabilities, self-determination is “a dispositional characteristic manifested as acting as the causal agent in one’s life” (Shogren et al., 2015, p.258). CAT is grounded in previous theoretical frameworks that have aimed to define self-determination construct, such as the Self-determination Theory (SDT, Deci & Ryan, 2012). While SDT explains why the person acts in a self-determined manner, specifically to fulfill their basic psychological needs, namely autonomy, competence and relatedness, CAT displays how the person engages in causal and self-determined action to fulfill those needs. In this sense, acting in a self-determined manner implies acting volitionally (i.e., making one’s own decisions and choices, setting goals on the basis of one’s preferences, and initiating action toward goals) and agentically (i.e., planning and acting toward attaining personal goals and evaluating the progress made to adapt the behavior accordingly). In turn, volitional and agentic actions nurture action–control beliefs (i.e., knowing one’s personal strengths and support needs, believing that one can reach self-set goals, and using knowledge about one’s skills, strengths, and resources to achieve those goals) (Shogren et al., 2015).

Some unique challenges commonly present in individuals with autism, including difficulties in social communication, interpersonal skills, and executive functioning, such as cognitive flexibility or inhibitory control, may hinder self-determination (Cheak-Zamora et al., 2020; Chou et al., 2017; Shogren et al., 2021; Tomaszewski et al., 2022). For example, difficulties in cognitive flexibility interfere with the ability to learn and integrate feedback from the environment and adapt to the demands of the context (Crawley et al., 2020), which can impede the ability to face problems and adapt to contingencies, both of which are necessary skills for agentic actions. Moreover, difficulties in cognitive flexibility have been linked to challenges in theory of mind and social communication (Memari et al., 2013), hindering the ability to achieve goals and navigate barriers that rely on effective social interaction. Thus, such difficulties affect the lives of people with autism, especially during their transition to adulthood, when they play an important role in employment status, postsecondary education options or independent living (Shogren et al., 2016). These specific challenges often adversely affect the self-determination of young adults with autism, who frequently exhibit lower self-determination scores compared to other quality of life (QoL) dimensions (Cuesta-Gómez et al., 2016) and other disability groups, such as those with intellectual or learning disabilities (Chou et al., 2017). Specifically, they reported difficulties with self-initiating actions, self-management skills, and problem-solving skills (Andrés-Gárriz et al., 2024b). Young adults with autism also express a lack of empowerment and difficulties in self-realization (Andrés-Gárriz et al., 2024b; Chou et al., 2017).

Given the uniqueness of the expression of self-determination in young adults with autism, tailored interventions are needed to address the specific support needs of this population. However, to the best of our knowledge, only four self-determination programs targeting young adults with autism have been developed: the *ACCESS Program* (Oswald et al., 2018), the *McGill Transition Support Program* (Nadig et al., 2018), the Family-centered Transition program developed by Hagner et al. (2012), and the recent neurodiversity-based self-determination program by McDonald et al. (2023).

The ACCESS program (Oswald et al., 2018) focuses on fostering self-determination, adaptive and social skills, and reducing stress and anxiety. It provides 19 sessions with social coaches who support participants throughout the intervention. This intervention yielded statistically significant improvements in self-determination as reported by the social coaches, with means 3.7 (95% CI 0.2–7.3) higher in the treatment group compared to the waiting list group (*p* =.04). The McGill Transition Support Program (Nadig et al., 2018) focuses on self-determination, working with others, and social communication. Based on an initial assessment of participants’ needs and interests in these three areas, the program provides 10 tailored sessions that are adapted to each specific intervention group. The program demonstrated positive effects on self-determination, particularly in interpersonal cognitive problem-solving, with the treatment group showing an average increase of 2 points (95% CI [0.082, 3.4], *p* =.04). However, the differences between groups did not reach statistical significance. The Family-centered Transition program (Hagner et al., 2012) is uniquely individualized, combining person-centered planning for the individual with autism and their support network with group sessions for family members. Participants of the program exhibited a significant improvement in self-determination, with mean scores increasing by 14.2 points after the intervention (*p* =.001). These three programs are based on the functional theory of self-determination (Wehmeyer, 1999), the theory that preceded CAT (Shogren et al., 2015). Finally, McDonald et al.‘s (2023) program is based on neurodiversity theory and aims to support young adults with autism (ages 18–34) in developing self-determination through a combination of group and individual sessions, integrating peer coaching and conflict resolution strategies. Although efficacy data are not yet available, a pilot study reported good appropriateness, high acceptability and good feasibility among participants and coaches. None of these programs have been delivered in Spanish to date.

Of these four programs, only the program by McDonald (2023) is based on the principles of the latest version of the self-determination framework, CAT (Shogren et al., 2015), despite the potential of this framework to guide programs that promote self-determination. A systematic review by Lindsay and Varahra (2021) found that CAT-based interventions, alongside other approaches, yielded significant improvements in self-determination outcomes for individuals with disabilities (e.g., Garrels & Palmer, 2020; Shogren et al., 2018; Shogren et al., 2020). While other models have also demonstrated efficacy in promoting self-determination, CAT offers several distinct advantages, as it incorporates key aspects that have gained prominence in recent years (Burke et al., 2024).

This theoretical model integrates advances from different theoretical frameworks, such as self-determination theory (Deci & Ryan, 2012), the Action-Control Theory (Little et al., 2002), and positive psychology, thus widening the perspective of self-determination and addressing previous misconceptions. It specifically builds on personal capacities for self-determination and considers the need for personalizing and systematizing environmental support (Burke et al., 2024; Shogren & Raley, 2022), thus standing as the most appropriate framework for leading interventions. Grounded in the current socioecological conception of disability, and in contrast to the other self-determination theoretical frameworks, CAT addresses some of the specific support needs of people with autism in the area of executive functioning. These needs include cognitive flexibility and inhibitory control (Shogren & Raley, 2022), which have a broad developmental window and can be improved through interventions, even during adulthood (e.g., Kim et al., 2021, 2023). Additionally, CAT inherently incorporates cultural considerations (Burke et al., 2024; Shogren & Raley, 2022), a critical aspect when promoting self-determination. This cultural sensitivity is especially relevant when comparing contexts such as the United States, where most self-determination interventions have been developed, and Spain, where the present research was conducted.

In Spain, where this study was conducted, few interventions have been developed to promote self-determination among people with disabilities, and none have comprehensively targeted young adults with autism. Existing programs are either designed for individuals with intellectual disability (Álvarez et al., 2022; Pascual-García et al., 2014) or focus narrowly on specific skills (Bonete et al., 2015). This limited scope may result in misconceptions about the self-determination construct because these programs often fail to address all dimensions of self-determination owing to their narrow focus (Shogren et al., 2015). Thus, considering (1) the impact of self-determination on QoL (e.g., Lachapelle et al., 2005) and postsecondary outcomes (Shogren & Shaw, 2016), (2) the need of people with autism to improve their self-determination-related skills (Cheak-Zamora et al., 2020; Chou et al., 2017), (3) the lack of updated intervention programs rooted in CAT, and (4) the lack of available intervention programs in Spain, where this study was conducted, this research seeks to address these issues by providing a flexible, contextually adapted program rooted in CAT to promote self-determination-related skills while addressing common support needs in individuals with autism, such as executive function, social communication, and social skills. Thus, the objective of the present study was to evaluate the effectiveness, implementation, and acceptability of the TEAM_YOUNG ADULTS program by answering the following research questions:

- RQ1. Was the program implemented appropriately (i.e., implementation)?
- RQ2. Is the program effective in improving the overall self-determination (or some dimensions of it) of Spanish young adults with autism (i.e., effectiveness)?
- RQ3. Was the program well accepted by the participants (i.e., acceptability)?

## Methods

### Design

**Fig. 1:**
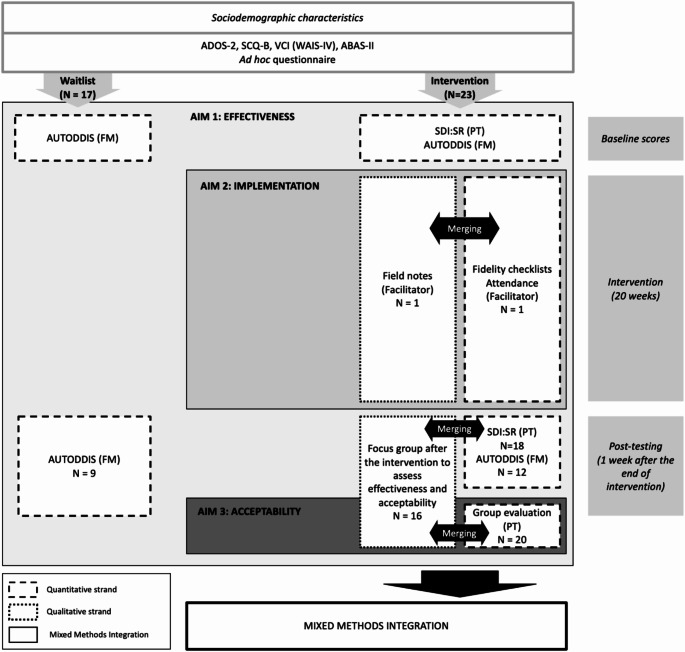
Mixed methods intervention design. *Note*. This process was followed for each intervention group, up to four times. PT = Participants; FM = Family members; ADOS-2 = Autism Diagnostic Observation Scale; SCQ = Social Communication Questionnaire; VCI(WAIS-IV) = Verbal Comprehension Index (Wechsler Adult Intelligence Scale-IV); ABAS-II = Adaptive Behavior Assessment System-II; SDI: SR = Self-Determination Inventory: Student Report

A mixed methods intervention design was used in which qualitative methods were embedded within a randomized controlled trial (RCT) (Creswell & Plano Clark, 2018; Fetters & Molina-Azorin, 2020). This type of design is becoming more prevalent in the field of developmental disabilities given its potential to ensure a more robust and comprehensive evaluation of complex interventions (Fàbregues et al., 2022). Mixed methods intervention designs can mitigate some of the limitations of RCTs when conducted alone, for example, by providing a more complete understanding of how and under what conditions an intervention works or by explaining differences in outcomes between participants (Johnson & Schoonenboom, 2016; Midgley, 2014). For the quantitative component of the mixed methods design, we employed a randomized controlled trial (RCT) with two parallel groups: an intervention group and a waiting list group. Participants were randomly assigned either to receive the intervention immediately (intervention group) or to be placed on a waiting list (waiting list group), with the latter receiving the intervention after the study period. This design enabled a controlled comparison of outcomes while ensuring that all participants eventually benefited from the program. For the qualitative component, we used focus groups and field notes to further expand and inform the quantitative results regarding the program’s effectiveness (e.g., to identify contextual barriers that help explain negative quantitative outcomes) and to gather complementary data about elements of program implementation and acceptability that were not examined in the quantitative component. Figure 1 shows the sequence of quantitative and qualitative methods employed, along with the evaluation objectives addressed by each. In reporting the mixed methods procedures and findings, we followed the Good Reporting of a Mixed Methods Study (GRAMMS) guidelines (O’Cathain et al., 2008). The RCT adhered to the World Health Organization’s (WHO, 2005) guidance for the conduct of clinical trials and the study complies with the CONSORT 2010 (Schulz et al., 2010) guidelines for reporting clinical trials.

### Setting

The study was conducted online, due to COVID-19 restrictions, from October 2020 to July 2022. Patients were recruited from two centers in Catalonia, Spain: a public mental health center for adults at the Hospital of Mataró and the Friends Foundation, which focuses on academic support for youth with autism. These centers were selected for two reasons. First, both services needed to provide support to young adults with autism that they could not otherwise deliver; second, for convenience, since the first two authors (C.A.G. and N.F.H.) were already familiar with these services.

### Participants

To participate in the study, young adults had to be aged between 17 and 30 years, currently engaged in one of the two aforementioned organizations, previously diagnosed with autism spectrum disorder according to DSM-5 criteria (American Psychiatric Association [APA], 2013) or Asperger syndrome according to DSM-IV (APA, 1994) by a mental health professional, and have a verbal comprehension index higher than 70 on the Wechsler Adult Intelligence Scale-IV (WAIS-IV; Wechsler, 2012) to ensure good comprehension of the program. The exclusion criteria were no spoken language or a condition that could significantly interfere with group functioning, preventing them or others from benefiting from the intervention. Examples of such conditions include acute psychotic disorders or challenging behaviors. Participants with other conditions, such as anxiety disorders or depression, were not excluded from the study.

### The TEAM_YOUNG ADULTS Program

The TEAM_YOUNG ADULTS program is a self-determination intervention specifically tailored for young adults with autism (17–30 years old), rooted in the Spanish context. It was created by the authors of this study and is based on the McGill Transition Support Program (Nadig et al., 2018), the *self-determined career design model* (SDCDM; Wehmeyer et al., 2003), and the principles of CAT (Shogren et al., 2015), which consider support in executive functioning. The intervention is structured in weekly sessions that provide psychoeducation in a flexible format. Personal experiences, opportunities, and problems are incorporated into psychoeducation sessions to guide participants in achieving their goals. For example, sessions have a designated time for psychoeducation on different topics, such as problem-solving, assertiveness, conflict resolution, barriers to and support for change, among others. Then, activities are conducted that relate to psychoeducation topics and build on the personal experiences of young adults. Thus, psychoeducation is implemented through activities during the sessions; real problems, challenges and strengths of the participants when possible; and homework assignments between sessions. If participants do not have real experiences to share, fictional situations are offered by the facilitators. The level of difficulty of the activities can be adapted, with different levels of complexity and support for each to suit the characteristics of the participants. Simpler situations and examples were provided for those participants with lower IQs. More detailed information about the program sessions and cultural adaptations can be found in the pilot study (Andrés-Gárriz et al., 2024a). The program can be delivered either online or in person. Owing to the limitations of COVID-19 in Spain, this study was conducted online. The intervention consists of 20 sessions, with one 1.5-hour session per week, divided into the following four modules: (1) *beginning and basic skills*, which provide support and teach skills directly related to the specific support needs common to people with autism; (2) *self-determination*, which provides lessons on self-determination-related skills on the basis of the participants’ specific goals; (3) *working with others*, which includes sessions on how to resolve conflicts with others; and (4) *closure*, the final module, which includes an evaluation of the progress made and a farewell session (see Table 1). A manual detailing the psychoeducation sessions is available upon request from the authors.

**Table 1:** Modules, sessions, and objectives of the TEAM_YOUNG ADULTS program

Module	*N*	Name of the session	Objectives
Beginning and basic skills	1	We start!	Introduction to the program Stablishing the rules for group functioning Meeting people from the group and the facilitators
	2	Decide, act and believe	To know the main goals of each participant to work on them over the program and to talk about different self-determination-related skills
	3	Abilities for the group	Communication styles What helps us to communicate with each other Understand or tolerate different perspectives
	4	Understanding the context	Learn to differentiate different contexts Learn to adapt the behavior depending on the context Identify how the context influences our decisions
	5	Good colleagues (and not that good ones)	Tolerance to different people Learning to differentiate good colleagues from not so good colleagues Learning to work with people you dislike
Self-determination	6	I know myself better	Identify personal preferences and interests Be aware of our strengths Learn to use interests and strengths to achieve our goals as well as to be aware of our limitations
	7	My goals	Learn to operatize goals Identify personal goals to work during the program Find other participants with goals in similar areas of their lives to support each other during the attainment of goals
	8	My plan	Thinking about steps to take to achieve the goals of each participant Think about possible barriers they may face and how to solve them
	9	My decisions 1	Learn to evaluate decisions Consider interests, strengths and values when making important decisions Learn a process to think about different possibilities and then decide based on a reflected process
	10	My decisions 2	
	11	What a problem!	Learn skills to solve problems How to face contingencies How to adapt goals and your behavior to different situations
	12	Obstacles and barriers	Identify possible obstacles and barriers that they can encounter in the process of attaining their goals Think about ways to solve such barriers
	13	Help!	Identify people who form our support network How to ask for help Share the process and sharing strategies
	14	Self-advocacy 1	Learning basic human rights Learning to stablish limits Learning assertiveness techniques
	15	Self-advocacy 2	
Working with others	16	Oh, conflicts… 1	Learning to identify conflicts and different styles to face them Learning strategies to prevent or solve conflicts
	17	Oh, conflicts… 2	
	18	Teamwork	Learning the added value of teamwork Identify how to contribute to a group Learning to value the strengths of others and our own
Closure	19	The path that we have walked together	Self-evaluation of the goals and the steps made to attain them. Analysis of the strategies applied by participants, accomplishments and self-determination Process of self-regulation: identifying if they need to change strategies, if goals were achieved, need further steps or need to be changed.
	20	Goodbye	Finish the group doing an in-person or online activity together

### Data Collection and Measures

#### Quantitative Data

Quantitative data were collected for each participant and their family members via self- and proxy-reported questionnaires at baseline and one week after program completion.

To assess the characteristics of the participants, the Autism Diagnostic Observation Scale–Second Edition (ADOS-2: Module 4; Lord et al., 2015), the Social Communication Questionnaire–Part B (SCQ-B; Rutter et al., 2019), the Wechsler Adult Intelligence Scale-IV (WAIS-IV: Verbal Comprehension Index; Wechsler, 2012) and the Adaptive Behavior Assessment System-II (ABAS-II; Harrison & Oakland, 2003) were used (more details on these measures can be found in Supplementary Material 1).

The Spanish version of the Self-Determination Inventory: Student Report (SDI: SR) (Mumbardó-Adam et al., 2018) was used to measure perceived self-determination in young adults with autism. On the basis of the principles of the CAT (Shogren et al., 2015a, b), the SDI: SR measures the essential characteristics and dimensions of self-determined action: volitional action (decide), agentic action (act), and action–control beliefs (believe). It is a self-report measure for youths between the ages of 13 and 22 years, with or without disabilities. The questionnaire has a good construct and acceptable concurrent validity, and the internal consistency of the three dimensions of self-determination ranged from good to excellent (α = 0.815 to α = 0.911) (Mumbardó-Adam et al., 2018). The Cronbach’s alpha for the sample in this study indicates high internal consistency (α = 0.959).

The AUTODDIS scale was used to measure self-determination from the family members’ perspective (Verdugo et al., 2021). This scale assesses self-determination in youth and adults (11 to 40 years old) with an intellectual disability through external informants. It consists of 46 4-point Likert-type items based on the degree of agreement. These items are arranged into three sections on the basis of the CAT: volitional actions, agentic actions, and action–control beliefs (Shogren et al., 2015a, b). The internal consistency of these sections was excellent (α = 0.948 to α = 0.962), as was that of the entire scale (α = 0.983), and there was also evidence of validity. The Cronbach’s alpha for the sample in this study indicates high internal consistency (α = 0.971).

The acceptability of the program was assessed using an ad hoc questionnaire consisting of five questions that could be answered from 1 to 10 and one closed-ended question (yes or no answer). The participants were asked to rate their satisfaction with the program, the quality of the materials and information, the facilitators, the perceived importance of the content, and the ability of the program to help them think and find solutions to problems. A final question asked whether they would recommend the program to others with similar support needs.

The fidelity of the implementation was measured using fidelity checklists. Each session had its own fidelity checklist, which assessed the implementation of the instructional sequence and program content. The fidelity checklists were completed by the facilitators at the beginning of the sessions to ensure rigorous implementation and at the end of each session to collect data on the consistency of the delivery of the program. The attendance of the participants who received the intervention or reasons for their absence was registered after each session.

#### Qualitative Data

Four focus groups were conducted by the first author after the completion of the intervention and before the administration of the postintervention quantitative measures. Sixteen of the 20 participants who completed the program agreed to participate in the focus groups. The participants in the four focus groups were the same as those in each of the four intervention groups. The questions focused on the perceived effectiveness of the program in improving self-determination and the participants’ views on its acceptability (see Supplementary Materials 2). The four focus groups ranged from 90 to 120 min and were conducted online using Google Meet.

To assess the implementation of the program qualitatively, field notes were collected by the intervention facilitators after each session. Field notes followed a narrative structure and were used to reflect on the intervention’s implementation, including barriers, facilitators, and potential future improvements. The facilitators documented any contingencies during the development of the session, the activities that worked better, and any aspects of the setting, structure, materials, or participants, among other factors, that influenced the program delivery.

### Data Analysis

#### Quantitative Analysis

Due to the small sample size, nonparametric tests were conducted. The Mann‒Whitney U test for independent samples was used to compare changes in self-determination between the group that completed the program and the waiting-list group. The Wilcoxon signed-rank test for dependent samples was used to examine changes in the experimental and control groups from the initial assessment to the postintervention assessment. In both cases, a significance level of 95% (*p* <.05) was used. Statistical analyses were performed with SPSS version 29.0. We calculated the r effect sizes to measure the strength of associations between variables. Effect sizes were interpreted based on Cohen’s guidelines, where *r* =.10 represents a small effect, *r* =.30 a medium effect, and *r* =.50 represents a large effect (Cohen, 1988).

#### Qualitative Analysis

The focus groups were videotaped and transcribed verbatim. Thematic analysis was used to analyze the focus group data in Atlas.ti Mac (version 9.1.3), following the six-step protocol established by Braun and Clarke (2006). C.A.G. familiarized herself with the data by reading the transcribed interviews and then performed initial inductive coding using a line-by-line coding approach, with codes reviewed by the last author (C.M.A.). C.A.G. then combined codes of similar content to generate themes and subthemes, which were subsequently further developed and revised by her and C.M.A. At this point, themes related to program effectiveness were generated, taking into consideration the construct of self-determination as defined by CAT (Shogren et al., 2015a, b) and its dimensions. In the final phase of the analysis, C.A.G. and C.M.A. refined and named the themes and resolved any disagreements by consensus.

#### Mixed Methods Integration

Following the completion of the quantitative and qualitative analyses, mixed methods integration was performed through merging (Fetters et al., 2013). Side-by-side joint displays, visual tools that facilitate the systematic integration of the two databases, were used to bring together both types of findings (Guetterman et al., 2015). Specifically, for each evaluation objective, the quantitative and qualitative findings were juxtaposed and compared to determine the extent to which they agreed, disagreed, or expanded or complemented each other. As a result of this procedure, we were able to generate meta-inferences that reflect unique mixed methods insights into the evaluation of the intervention that could not have been obtained using either quantitative or qualitative methods alone (Younas et al., 2023). These unique insights are explicitly reported in both the Results and Discussion sections.

### Ethical Considerations

The study was conducted in accordance with the Declaration of Helsinki and was approved by the Ethics Committee of Ramon Llull University and the Hospital of Mataró (CEIm_76/20, October 10, 2020). This trial is registered at www.clinicaltrial.org (NCT05938751). Written consent was obtained from young adults with autism. Parents provided informed consent for participants who were minors (under 18 years of age in Spain) after obtaining prior assent from the young adult. All participants were informed of the study and the option to discontinue participation without any repercussions at any time.

## Results

### Enrollment and Randomization

**Fig. 2:**
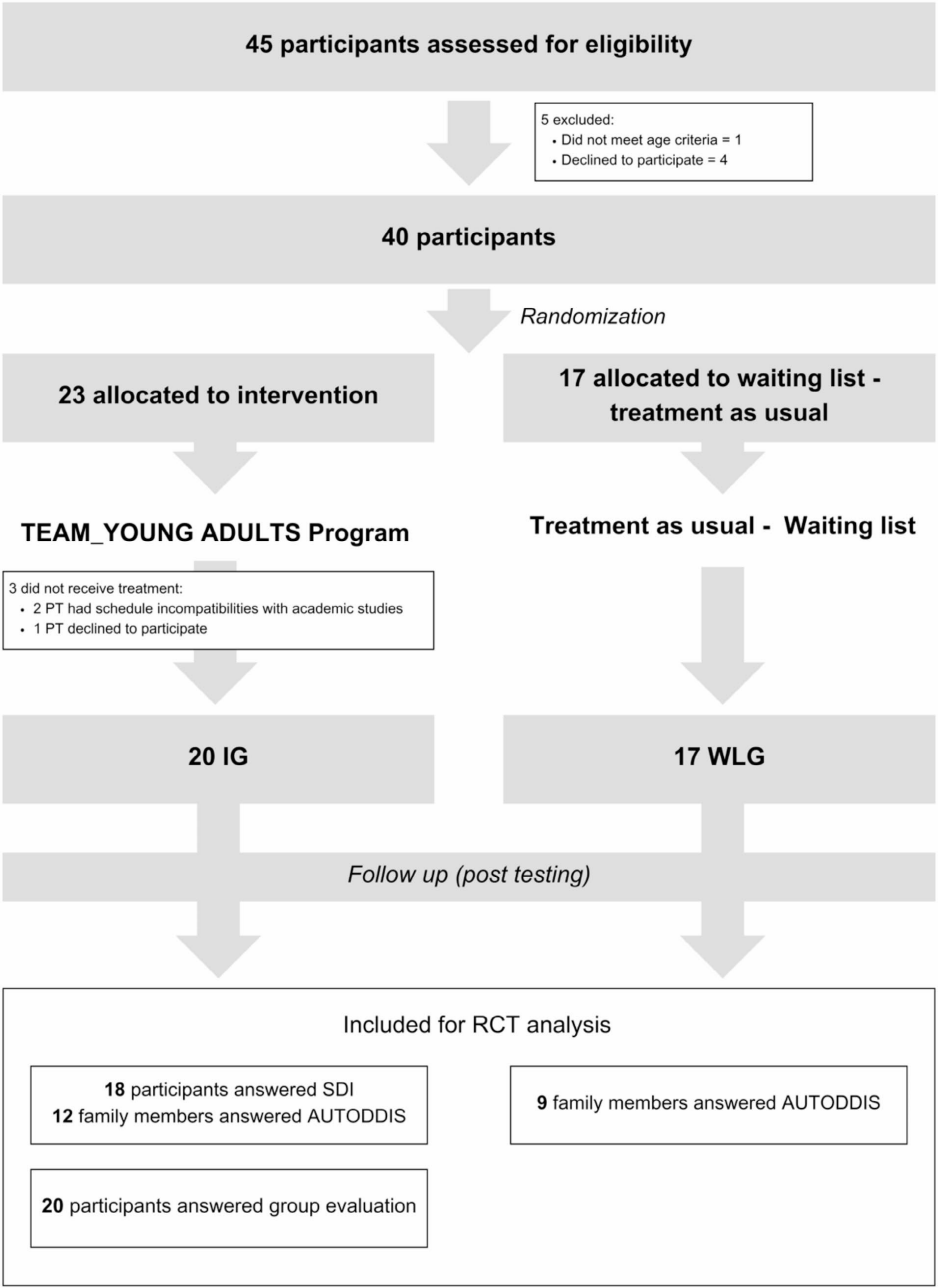
Participant flow chart. *Note*. PT = Participants; IG = Intervention group; WLG = Waiting list group; FGs = Focus groups; SDI = Self-Determination Inventory: Student Report; AUTODDIS = AUTODDIS Scale

Mental health professionals from the Child and Youth Mental Health Center and the Adult Mental Health Center of the Hospital of Mataró and Friends Foundation contacted the participants. Those who expressed interest in the study were subsequently referred to C.A.G., who assessed their eligibility and provided detailed information about the study objectives and procedures. If a young adult agreed to participate in the study, an online meeting was scheduled. For participants under 18 years of age, the legal age of adulthood in Spain, the parents signed an informed consent form with the previous assent of the young adult. Of the 45 individuals contacted, 40 agreed to participate. Four cohorts were analyzed over a two-year period. Participants who agreed to participate and met the inclusion criteria at each attempt were randomly assigned to either the intervention (23 participants) or waiting-list group (17 participants). Randomization was stratified by sex (i.e., male, female, or nonbinary). There were four recruitment attempts, each resulting in an intervention group, in January 2021, March 2021, November 2021, and January 2022. A total of 20 participants completed the intervention and participated in the final focus group discussion. Among these participants, 18 completed the follow-up questionnaire. There was high attrition in the waiting-list group, where only 9 out of 17 caregivers completed the follow-up measures. Further information on the recruitment process is shown in Fig. 2. Blinding participants and facilitators was not feasible due to the nature of the intervention. Similarly, it was not possible to blind participants and researchers during the focus groups, or the researchers performing the qualitative analysis. However, for the quantitative analysis of the intervention’s effectiveness, one researcher involved in the quantitative analysis was kept blinded to group allocation to minimize bias.

### Participant Characteristics and Baseline Data

Participant demographics, including age, gender, living situation, educational or employment status, education level, co-occurring conditions, psychological treatment, and medication use, showed no significant differences between the intervention and waiting list groups. Additionally, characteristics related to autism symptoms, verbal comprehension, and adaptive behavior were also comparable between the two groups (see Table 2).

**Table 2:** Participant demographics and baseline scores on outcome measures

	Intervention (*N* = 18) *N*(%) or M(SD), range	Waiting list (*N* = 9) *N*(%) or M(SD), range	Statistical	gl	*p*
*Demographics*					
Age	20.11(2.22), 17–25	19.78(3.35), 18–28	*U* = 59.000		0.246
Gender			χ^2^ = 0.529	2	0.767
Men	11(61.1%)	6(66.7%)			
Women	6(33.3%)	2(22.2%)			
Non-binary	1(5.6%)	1(11.1%)			
Living situation			χ^2^ = 0.519	1	0.471
Living with family members	17(94.4%)	9(100%)			
CRAE	1(5.6%)	0			
Study/employment status			χ^2^ = 3.671	3	0.299
Studying	14(77.8%)	5(55.6%)			
Working	1(5.6%)	1(11.1%)			
Not studying nor working	3(16.7%)	3(33.3%)			
Education level			χ^2^ = 5.884	4	0.208
Primary education	1(5.6%)	1(11.1%)			
Secondary education	3(16.7%)	5(55.6%)			
High school	5(27.8%)	2(22.2%)			
Vocational training (medium grade)	8(44.4%)	1(11.1%)			
Vocational training (high grade)	1(5.6%)	0			
Co-occurrent conditions			χ^2^ = 5.727	7	0.572
Anxiety	2(11.1%)	0			
OCD	1(5.6%)	1(11.1%)			
ADHD	6(33.3%)	2(22.2%)			
BPD	1(5.6%)	0			
PTSD	1(5.6%)	0			
High intellectual abilities	1(5.6%)	0			
Depression and anxiety	0	1(11.1%)			
None	6(33.3%)	5(55.6%)			
Psychological treatment (yes)	5(27.8%)	0	χ^2^ = 3.068	1	0.080
Medication (yes)	10(50%)	4(44.4%)	χ^2^ = 0.000	1	1.000
*Characteristics*					
ADOS-2	9.22(2.29), 7–15	7.78(0.833), 7–9	*U* = 56.500		0.102
SCQ-B	17.89(6.96), 2–32	15.78(6.48), 8–27	*U* = 65.500		0.247
VCI (WAIS-IV)	106.00(16.79), 80–136	95.78(12.05), 75–114	*U* = 57.000		0.119
ABAS-II	68.38(14.20), 53–100	75.33(7.25), 68–90	*U* = 63.000		0.266
Conceptual index	63.83(12.42), 54–91	67.56(14.25), 54–88	*U* = 72.500		0.647
Social index	69.62(11.31), 57–96	73.67(9.75), 65–94	*U* = 70.500		0.457
Pragmatic index	66.17(12.71), 54–95	70.56(10.50), 59–91	*U* = 66.000		0.438
General adaptive behavior					
*Baseline self-determination*					
AUTODDIS					
Volitional action	36.00(8.28)	40.33(8.53)	*U* = 71.500		0.625
Agentic action	32.83(13.62)	34.00(11.36)	*U* = 78.000		0.877
Action-control beliefs	46.33(11.36)	49.78(8.54)	*U* = 71.500		0.625
Total	115.17(31.71)	124.11(25.03)	*U* = 80.500		0.979

Note ADOS-2 = Autism Diagnostic Observation Scale; SCQ = Social Communication Questionnaire; VCI(WAIS-IV) = Verbal Comprehension Index (Wechsler Adult Intelligence Scale-IV); Psychological treatment = Individual therapy outside of the program

Baseline self-determination scores on the AUTODDIS scale (Verdugo et al., 2021) indicated similar levels of overall self-determination, as well as in the three dimensions, for both the intervention and waiting list groups (see Table 2).

#### RQ1.

Was the Program Implemented Appropriately (i.e., Implementation)?

#### Quantitative Results

High implementation fidelity was demonstrated for all groups per session and for the curriculum module, with implementation fidelity ranging from 97.25 to 98.77% for each group. Attendance was recorded for each session. The average attendance rate was 84.04%, ranging from 50 to 100%. Most participants attended over 80% of the sessions, whereas only one participant attended half of the sessions. The reasons for not attending the sessions included medical appointments, studying for exams, and forgetting the group date after a holiday break.

#### Qualitative Results

Four themes and seven subthemes were identified regarding the program’s implementation (see Table 3 for a summary of themes and subthemes and Supplementary Material 3 for more details).

**Table 3:** Themes and subthemes of the effectiveness, implementation, and acceptability of the program

Dimensions	Themes	Subthemes
Effectiveness	Changes in volitional actions	Autonomy
		Self-initiation
	Changes in agentic actions	Self-direction
		Self-regulation
	Changes in action-control beliefs	Control expectancy
		Empowerment
		Self-realization
	Facilitators for change	Personal attitudes and interests
		Environmental supports
		People of support
	Barriers for change	Barriers to volitional action
		Barriers to agentic action
		Barriers to action-control beliefs
Implementation	Format	Structure
		Setting
		Materials
	Content	Content evaluation
		Content application
	Challenges	Participants’ challenges
		Participation and group cohesion challenges
	Improvements	-
Acceptability	Usefulness of the program	-
	Advantages of the group format	-
	Suggestions for improvement	-

Note Participants reported effectiveness and acceptability. The facilitators of the groups reported implementation

Regarding the format of the program, the intervention was found to be proficiently structured. Having an introductory part in each session to talk about experiences and steps toward goals was also considered helpful. Facilitators stated, “The initial part to see how the week went works very well. It works as a follow-up to the objectives.” In addition, small and large group activities were positively evaluated, and depending on the group, one format or the other was preferred. The length of the sessions was good for most participants. Two participants felt tired or had problems focusing at the end. However, for some of the groups, not all activities could be completed in 1.5 h because the participants were actively engaged in the activities, but these activities were finished at the beginning of the next session. In one group, an additional in-person social gathering was held to create better group cohesion. Finally, the materials were useful for following the sessions.

Regarding the content of the intervention, the evaluation of most sessions was excellent, with a high level of acceptance for the activities. The program made it possible to share experiences among the participants and provide new perspectives. For example, during the first group, one of the facilitators noted, “P1 brings a new perspective to P4 and helps to find possible solutions to her problem.” The use of the participants’ experiences was also encouraged, thus allowing the application of the program content to real-life situations.

Challenges were also identified. Young adults experienced difficulties in generalizing from theory to practice, implementing decisions and finding alternative solutions to problems. One participant exhibited strong avoidance of insights, which made him uncomfortable discussing goals and the future. Furthermore, the profile of certain participants presented some challenges that were difficult to address in the program and during group sessions, for example, in the case of participants with cooccurring conditions, such as posttraumatic stress disorder (PTSD) or borderline personality disorder (BPD). A facilitator wrote, “P17 would need weekly individual meetings and specialized treatment for PTSD.” Additionally, one group experienced challenges with group cohesion at the beginning of the program. This also led to reduced participation, making the program more difficult to implement. After the in-person session, group cohesion improved.

Finally, the participants proposed several suggestions for improvement, such as completing the questionnaires as homework assignments to avoid long waits during the session or adding more small group activities and additional levels of complexity in the examples. The inclusion of a booklet with written materials would also provide good support to the participants. Finally, a more thorough analysis of the participants’ profiles could enhance group cohesion and improve the adaptation of the sessions. One facilitator stated, “In future groups, it would be necessary to better assess the profile of the participants according to their abilities and conditions to better match the members of the groups.”

##### RQ2.

 Is the Program Effective in Improving the Overall Self-determination (or Specific Dimensions of it) of Spanish Young Adults with Autism (i.e., Effectiveness)?

#### Quantitative Results

Findings regarding the effectiveness of the intervention in improving self-determination indicated a significant difference in agentic action perceived by participants (*Z* = -2.516, *p* =.010, *r* =.60) and their families (*Z* = -2.451, *p* =.014, *r* =.71), which was greater in both cases after program completion. These findings indicate that participants and their caregivers perceived an improvement in young adults’ ability to plan and act toward their goals, attain them, adapt to contingencies, and evaluate their progress. Overall self-determination, as perceived by family members, increased significantly after the program (*Z* = -2.049, *p* =.040, *r* =.59). The participants’ other self-determination measures did not significantly change; however, the mean scores for action–control beliefs and general self-determination increased after the program (see Table 4).

**Table 4:** Intra and intergroup comparisons of self-determination

Outcome		Intervention (*N* = 12)		Intragroup comparison				Waiting list (*N* = 9)		Intragroup comparison			Intergroup comparison		
	*N*	Pre M(SD)	Post M(SD)	Z	*p*	*r*	*N*	Pre M(SD)	Post M(SD)	Z	*p*	*r*	U	*p*	*r*
Proxy-report: AUTODDIS															
Volitional action	12	36.00(8.28)	39.33(9.52)	-1.904	0.057	0.550	9	40.33(8.53)	40.22(6.16)	-0.105	0.916	0.004	53.50	0.972	0.008
Agentic action	12	32.83(13.62)	37.17(13.56)	-2.451	0.014*	0.710	9	34.00(11.36)	30.67(8.59)	-1.782	0.075	0.590	39.00	0.310	0.233
Action-control beliefs	12	46.33(11.36)	49.50(10.63)	-1.684	0.092	0.490	9	49.78(8.54)	47.67(7.35)	-1.483	0.138	0.490	53.00	0.972	0.155
Overall self-determination	12	115.17(31.71)	126.00 (32.48)	-2.049	0.040*	0.590	9	124.11(25.03)	118.56(20.38)	-1.153	0.249	0.380	49.00	0.754	0.077
Self-report: SDI: SR															
Volitional action	18	73.00(17.82)	72.67(18.60)	-0.521	0.602	0.120	-	-	-	-	-		-	-	
Agentic action	18	67.72(20.47)	75.39(17.12)	-2.561	0.010*	0.600	-	-	-	-	-		-	-	
Action-control beliefs	18	69.83(18.67)	72.94(19.68)	-1.161	0.246	0.270	-	-	-	-	-		-	-	
Overall self-determination	18	210.56(54.86)	221.00(50.81)	-1.894	.058	.450	-	-	-	-	-		-	-	

No significant differences were found between the self-determination outcomes of the intervention and waiting-list groups. No significant differences in the dimensions related to self-determination were noted between the two groups (Table 4).

#### Qualitative Results

Five themes and 13 subthemes were identified regarding the program’s perceived effectiveness (see Table 3 for a summary of themes and subthemes and Supplementary Material 3 for more details).

The participants referred to improvements in their volitional actions, which is the ability to set goals, decide by oneself, and initiate actions toward goals. Most young adults reported being able to make independent decisions on the basis of their preferences and past experiences, which enhanced the quality of their decisions. Some participants expressed that they were acting toward their goals and were beginning to take action. For example, Participant 1 noted, “I see that now I can not only make decisions, which was already difficult for me, but also put them into practice. Now, it seems to be happening in a much more fluid way.” On the other hand, two participants mentioned difficulties in self-initiation, such as not sending CVs to search for a job or not acting because of emotional dysregulation. Participant 10 stated, “Sometimes I say I’m going to do something, but in the end, I don’t do it. I don’t feel well emotionally to do so.”

Young adults noted several changes in their agentic actions (related to planning and attaining goals, evaluating the progress made toward goals, and the ability to adapt to contingencies). The participants reported improved self-advocacy skills and the ability to act and defend their rights and needs. Improved planning, goal attainment, and problem-solving skills were also noted. For example, Participant 12 expressed, “If I see that I’m overwhelmed, I stop for a moment, go watch a show or something, and then I come back [to work on her goal].” In addition, their self-monitoring skills were also enhanced, as they were able to modify their actions toward reaching their goals on the basis of their experiences.

Slight improvements in action–control beliefs (related to self-knowledge of strengths and difficulties, believing in oneself, and knowing the support network and how to use it to achieve goals) were also reported. The participants expressed asking more for help, using support to achieve goals, and being able to evaluate their experiences. They described increased self-acceptance, confidence in their own decisions and abilities, and the ability to face challenges and achieve their goals. For example, Participant 4 stated, “I have better self-esteem.” Finally, they demonstrated a better understanding of their strengths and weaknesses. Some participants also mentioned their ability to use their personal strengths to achieve their goals.

Facilitators of change included environmental support, personal attitudes and interests, and supportive individuals. Increased opportunities and exposure to situations helped participants practice their skills, gain experience, and begin making decisions independently. Participant 8 reported, “I have tried to be in situations and do things that I didn’t think I would be able to do before.” In addition, certain personal attitudes and interests, such as motivation and setting goals related to their interests, seemed to promote goal attainment, thus assisting participants in achieving their goals. Striving to achieve goals was also identified as an attitude that facilitated agentic actions, especially when fewer motivational steps needed to be taken to achieve a larger goal. The main sources of support reported by the youth were their mothers, other family members, friends, and professionals, such as psychologists and educators.

Barriers to change were identified in the three dimensions of self-determination. A lack of motivation or interest was one of the most prevalent barriers to volitional actions. For example, Participant 6 noted, “Nothing motivates me.” Viewing activities or actions as less important and avoiding those perceived as difficult also hindered motivation.

In terms of barriers to agentic actions, some were contextual barriers, such as having to wait to attain long-term goals and a lack of support from individuals, which hindered goal attainment. Other barriers included personal challenges, such as inconsistency in following the established plan to achieve goals, as well as autism-related support needs, such as difficulties in cognitive flexibility and adjusting to environmental demands. For example, Participant 7 stated, “When I am faced with a problem that I don’t have the answer or the knowledge to solve, I don’t know what to do.” In addition, social difficulties contributed to social problems or hindered problem solving in some participants. Finally, some participants lacked awareness of their progress, denying the changes that the other participants and facilitators perceived.

Action–control beliefs also presented barriers. Three participants did not want to ask for help for different reasons, including fear of being criticized or a desire to complete all tasks without support. Additionally, two of these participants felt unable to achieve their goals. Some participants showed an external locus of control, whereas others expressed that they did not know how to apply their strengths to achieve their goals. For example, Participant 16 stated, “I don’t know how to use my strengths.”

##### RQ3.

Was the Program Well Accepted by the Participants (i.e., Acceptability)?

#### Quantitative Results

The acceptability results revealed that the participants gave good ratings to the program for overall satisfaction (M = 8.45, *SD* = 1.40), materials and information (M = 8.70, *SD* = 1.42), importance of content (M = 8.25, *SD* = 1.71), help with thinking and finding solutions to problems (M = 7.94, *SD* = 1.57), and excellent ratings to the facilitators (M = 9.10, *SD* = 1.33). 100% of the participants would recommend the program to others.

#### Qualitative Results

Three themes were identified regarding the program’s acceptability (see Table 3 for a summary of themes and subthemes and Supplementary Material 3 for more details).

The young adults with autism expressed that they had learned resources and skills and emphasized that they were able to share their experiences with others. They also perceived changes during the program and felt better able to adapt to contingencies. Overall, most participants provided positive evaluations of the program. In addition, the young adults expressed that doing the program in a group helped them feel supported through difficulties. For example, Participant 10 noted, “It is good to do it in a group because you see what other people think, and they also have problems like you.” Finally, one suggestion for improvement involved combining the group with individual sessions.

### Mixed Methods Integration

**Fig. 3:**
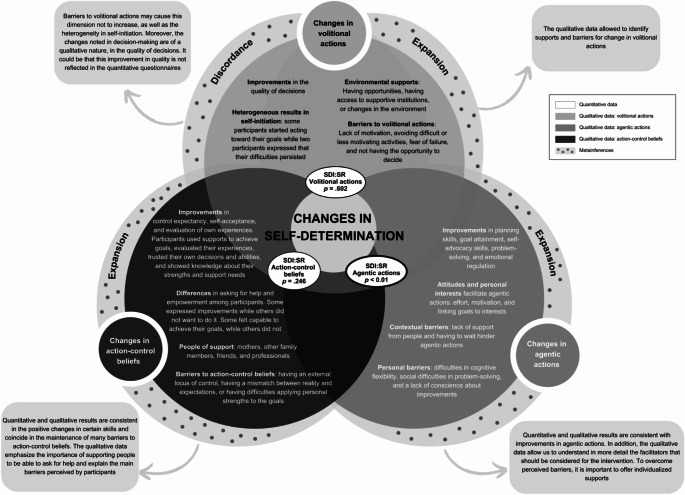
Joint display of the effectiveness of the intervention

Figure 3; Tables 5 and 6 show three joint displays that report the integration of the quantitative and qualitative results on program implementation, effectiveness, and acceptability. For program effectiveness, the quantitative findings were primarily enriched by the qualitative data in the three dimensions of self-determination. A divergence was noted only in volitional actions, where qualitative findings reported improvements that were not reflected in the quantitative results. The two strands complemented each other for program implementation. The qualitative data provided additional information about the program’s format, content, challenges, and improvements. Finally, the qualitative findings on acceptability expanded the information from the quantitative data, allowing for a better understanding of the most valued aspects of the program and suggestions for improvement.

**Table 5:**
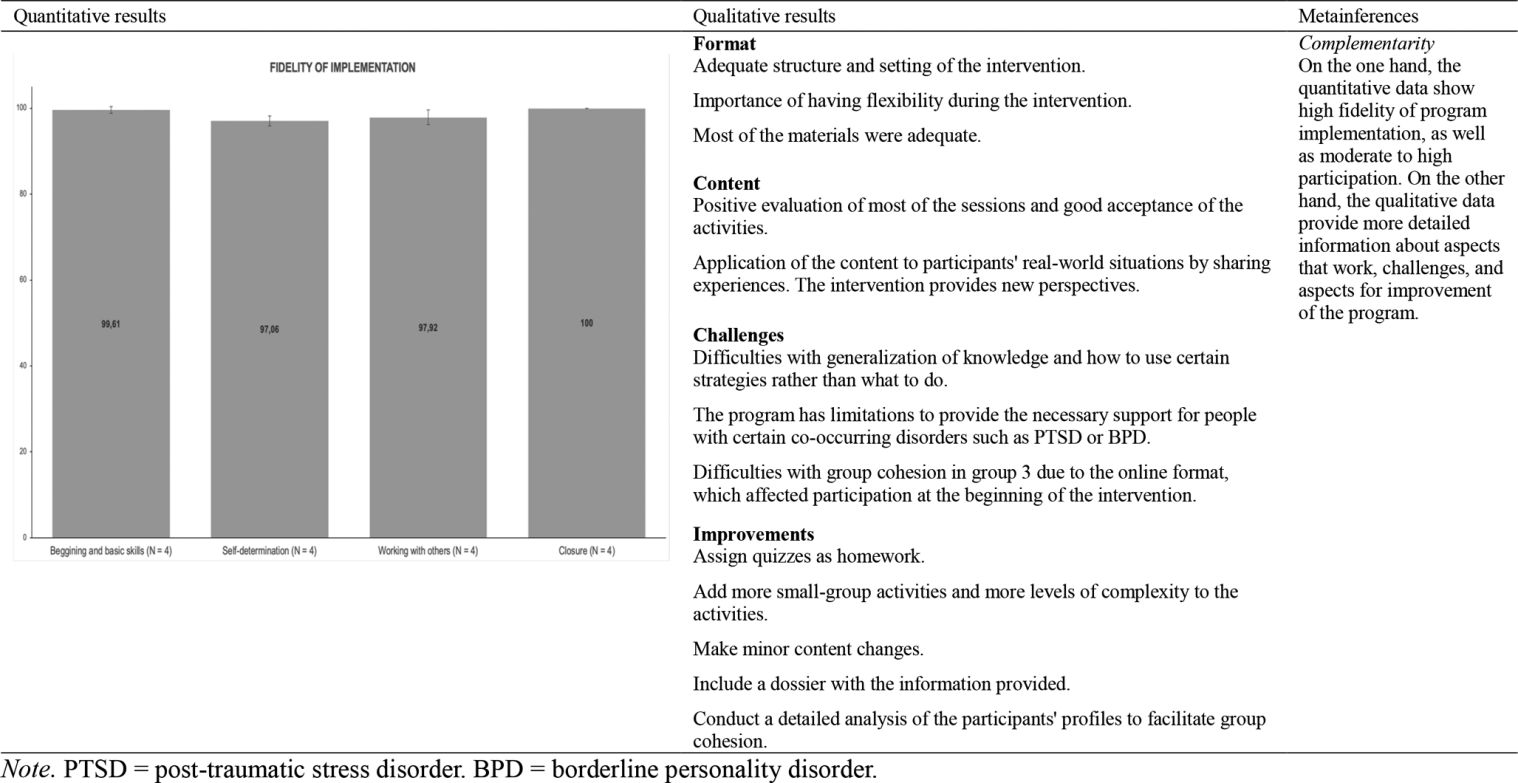
Joint display of the implementation of the intervention

**Table 6:**
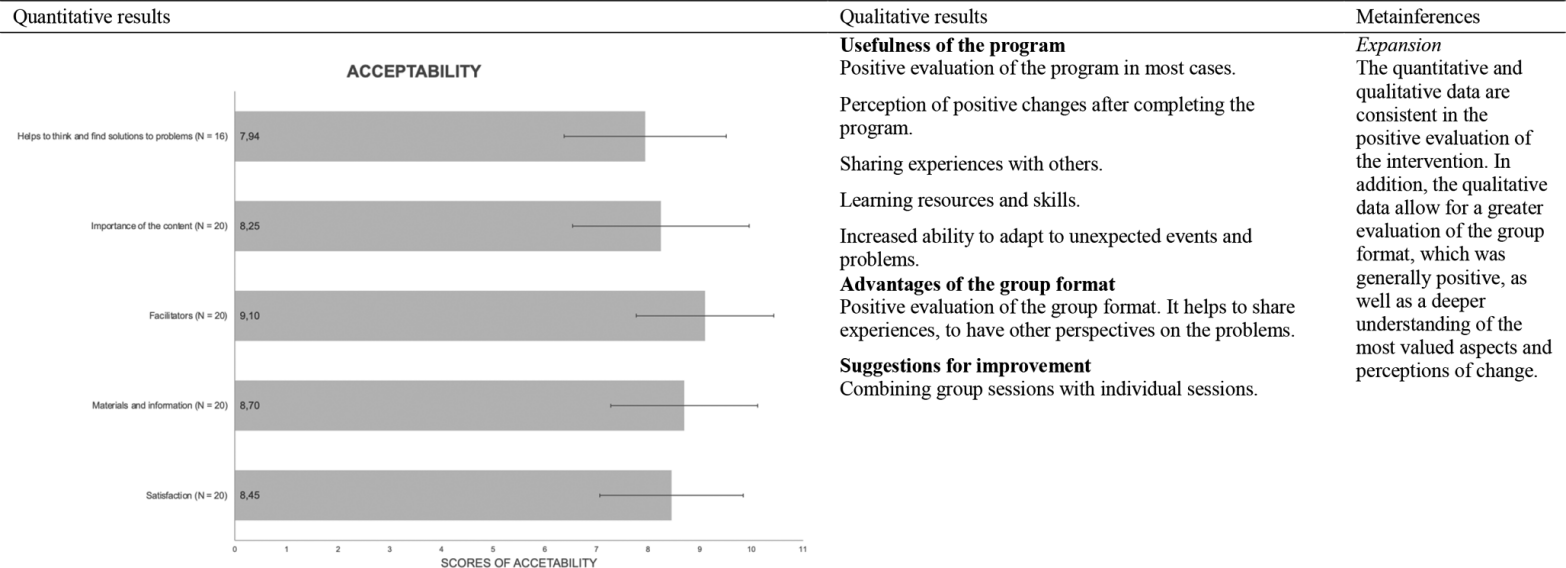
Joint display of the acceptability of the intervention

## Discussion

The main objective of the present study was to evaluate the implementation, effectiveness, and acceptability of a newly developed self-determination promotion intervention, namely, the TEAM_YOUNG ADULTS program, to better understand its potential to support young adults with autism without intellectual disability in the development of their self-determination-related skills. Integrating quantitative and qualitative data facilitated a comprehensive evaluation of the program, leading to a deeper understanding of the changes in self-determination experienced by the participants and offering precise insights into the challenges and suggestions for program enhancement.

The findings addressed all three research questions of the study. For RQ1 (*Was the program implemented appropriately (i.e.*,* implementation)?*), the results indicated that the program was effectively implemented, with qualitative data offering valuable insights into its strengths and areas for improvement. Regarding RQ2 (*Is the program effective in improving the overall self-determination (or some of its dimensions) of Spanish young adults with autism (i.e.*,* effectiveness)?*), while no significant differences were identified in self-determination between the intervention and waiting list groups, the program contributed to notable improvements in agentic actions, goal attainment, problem-solving, and self-regulation. Lastly, for RQ3 (*Was the program well accepted by the participants (i.e.*,* acceptability)?*), the program demonstrated high levels of acceptability among participants, who provided positive feedback on various aspects of the intervention.

### RQ1.

Was the Program Implemented Appropriately (i.e., Implementation)?

The combination of the fidelity checklists and the field notes yielded complementary findings. The high scores on the fidelity checklists indicated high fidelity of implementation, whereas the qualitative findings allowed for a deeper exploration of potential improvements and challenges that need to be considered in the future. Consistent with the observations of McDonald et al. (2023), our results suggest that more specialized and specific services are needed to support people with cooccurring conditions, such as PTSD, BPD, or obsessive‒compulsive disorders (OCDs). Another important challenge was group cohesion, particularly for Group 3 at the beginning of the intervention. Therefore, further analysis of the participant profiles is needed to create more homogeneous groups that may facilitate interaction (McDonald et al., 2023; Nadig et al., 2018). In addition, although the online format was beneficial for most participants because it facilitated their attendance, others preferred a face-to-face format. Future research should implement the program in a face-to-face format to explore its benefits and limitations. These aspects of improvement highlight the need for further flexibility to adapt the implementation of activities, format, and support to individual needs and preferences. Although our program was flexible and adapted the content and the pace to the goals and needs of the participants, more flexibility in the format could provide greater support.

### RQ2.

Is the Program Effective in Improving the Overall Self-determination (or Specific Dimensions) of Spanish Young Adults with Autism (i.e., Effectiveness)?

Overall, although an increase in self-determination was observed in the intervention group, no statistically significant differences were noted between the intervention and waiting-list groups. These findings are similar to those reported in the McGill transition support program (Nadig et al., 2018), where the total score of self-determination was higher in the intervention group than in the waiting-list group after implementation, but no significant changes were detected. Owing to the small number of caregivers who completed the posttest questionnaires, a larger sample size is needed to draw further conclusions about the intervention program’s impact.

However, the integration of quantitative and qualitative data highlighted positive changes in agentic actions, consistently with other studies in which skills related to agentic actions exhibited enhanced improvements (e.g., goal attainment, McDonald et al., 2023; e.g., social problem-solving, Nadig et al., 2018). These findings also align with one of the major recommendations of the CAT in adulthood: the promotion of problem-solving and self-advocacy skills related to agentic actions (Shogren & Raley, 2022). Importantly, this dimension had the greatest reported change after the intervention based on CAT. Moreover, the qualitative data highlighted several facilitators and barriers to agentic actions with implications for practice. Motivation was greater when goals were aligned with preferred interests, which facilitated goal attainment, in line with Winter-Messiers et al. (2007). Conversely, personal barriers, such as serious difficulties in cognitive flexibility, social difficulties in problem solving, or a lack of conscience about improvements, may indicate that the program might target those needs. For example, the proposal to combine group sessions with individual sessions could provide such individualized support, although there may be other strategies to improve program outcomes.

Neither volitional actions nor action–control beliefs significantly changed after the intervention, although the former increased in the intervention group according to the quantitative and qualitative data. An improvement in the quality of decisions, which could not be detected in the questionnaires, was reported, and some of the participants referred to self-initiation changes. The barriers identified based on the qualitative data may explain the lack of significant changes despite an improvement being detected. For example, the lack of motivation in intermediate steps to achieve a goal, avoidance of difficult activities or fear of failure hindered self-initiation. Considering that young people with autism show difficulties in self-initiation (Andrés-Gárriz et al., 2024b), they may need more intensive support in this area to show significant changes. Furthermore, barriers to volitional actions, such as a lack of motivation, may be linked to support the need for inhibitory control (Shogren et al., 2021). When confronted with unmotivating steps toward goals, individuals must be able to suppress their initial response to engage in a more motivating activity and focus on long-term goals by tolerating short-term frustration in the current unmotivating activity, a skill related to inhibitory control. Individuals with autism and inhibitory control tend to delay the initiation of action, and must thus be supported in natural environments, where they show these difficulties. Future research on this program should thus consider its application in community-based settings.

Action–control beliefs have emerged as the most resistant dimension to change because they involve beliefs and attitudes that require time, experience, and opportunities for development. Thus, the duration of the program may be insufficient to produce significant changes. Therefore, additional follow-up sessions or different forms of support are needed to further promote action–control beliefs in young adults with autism.

Notably, in our program, participants perceived their family members as supportive of self-determination, which aligns with the results of Roche Cárcel (2021) concerning the importance of family values in the Spanish population. In contrast, our results differ from those of other studies (e.g., McDonald et al., 2023; Nadig et al., 2018), where participants preferred that their relatives not be involved in the intervention process. These disparities underline the importance of considering cross-cultural differences in self-determination between the Spanish and U.S. contexts (as in other disability groups, e.g., Shogren et al., 2019) when developing interventions. Therefore, targeted interventions for families are crucial to complement self-determination programs such as TEAM_YOUNG ADULTS.

### RQ3.

Was the Program Well Accepted by the Participants (i.e., Acceptability)?

The integration of findings provided evidence of good acceptability of the program. The findings showed that the intervention program was well received by the participants. As a suggestion for improvement, some participants advised combining the group and individual sessions. The group format has been shown to have several benefits, such as providing social support, normalizing participants’ lived experiences, allowing for collaborative problem-solving, reducing feelings of loneliness, and promoting positive social experiences (Spain & Blaney, 2015). Additionally, the group format represents a cost-effective approach to the delivery of intervention programs in public health settings. Conversely, individual sessions would permit more intensive and tailored support. Thus, the combination of group and individual sessions, as seen in other programs such as McDonald et al. (2023), would be beneficial and should be considered in future implementations of the TEAM_YOUNG ADULTS program.

### Limitations

This study has several limitations. First, there was a high attrition rate, especially in the waiting-list group. The instrument used to compare self-determination between the intervention and waiting-list groups was a proxy report measure completed by the participants’ caregivers. The greatest attrition was observed in the waiting-list group, with 17 participants who completed the pretest questionnaires and 9 who completed the posttest. Future research should consider how to engage participants in the waiting-list group to improve their response rate to posttest questionnaires. Notably, financial incentives for participation are not commonly offered; thus, alternative strategies such as further follow-up or contact during the months of program implementation may represent a possible solution to keep caregivers engaged. Second, this study used convenience sampling of participants from two institutions, one of which is a public hospital, which may have introduced bias into the results. Notably, participants associated with the Adult Mental Health Center of the hospital in most cases had a cooccurring condition or challenging socioeconomic and family situations, which may have negatively influenced the results. However, this speaks in favor of the study results, as implementation of the program showed positive outcomes for participants with cooccurring conditions or challenging situations. Third, the self-determination measures used in this study are not well suited to our sample. The SDI: SR has been tailored to individuals with and without disabilities and adapted to the Spanish context (Mumbardó-Adam et al., 2018), but its psychometric robustness has not been studied in people with autism. Furthermore, it only targets students aged between 13 and 22 years. Conversely, although the AUTODDIS scale is suitable for the age of our sample (for people between 11 and 40 years), it was developed for people with intellectual disability. Fourth, one participant had a cooccurring condition of PTSD, and another participant also had BPD. These conditions may have impacted group functioning due to the higher level of support needed by these two participants. Fifth, the fidelity checklists were completed by the group facilitators, which may have introduced bias. Future studies should use an unbiased evaluator to assess fidelity. Nevertheless, the fidelity checklists that were used verified that various actions or activities were followed during the sessions, not the quality of implementation, so the risk of bias is limited. Sixth, conducting a blinded study was not feasible, which may have introduced potential bias. Blinding in educational interventions is inherently challenging (Styles & Torgerson, 2018), particularly when employing a mixed methods design (Boeije et al., 2015). Although it was not possible to blind participants or facilitators, we implemented blinding for the researcher conducting the quantitative analysis of the intervention’s effectiveness by concealing group allocation. This measure was taken to minimize the risk of bias and enhance the validity of the study.

## Conclusions

In this study, we presented a self-determination promotion program specifically tailored for young adults with autism, the TEAM_YOUNG ADULTS program, which is based on the CAT (Shogren et al., 2015a, b). The program did not show significant differences between the intervention and the waiting list groups but generated changes in agentic actions and the attainment of goals, problem-solving, and self-regulation. It was also well implemented, and the qualitative data allowed for further reflection on the strengths and limitations of the program. Finally, the program showed high acceptability among participants, who expressed a willingness to recommend the program to others and who provided positive evaluations of different aspects of the intervention. Furthermore, the mixed methods approach was effective in providing a more accurate and comprehensive understanding of the program’s effectiveness, implementation and acceptability.

Finally, and perhaps most importantly, this is the first empirical evidence of self-determination promotion in people with autism in Spain. Our program showed promising results and represented a preliminary step toward supporting individuals with autism in the Spanish context. Further research is needed to evaluate its effectiveness and to develop evidence-based interventions (Schalock et al., 2017). The development of programs to promote self-determination in individuals with autism is crucial for enhancing postsecondary outcomes (Shogren & Shaw, 2016; Shogren et al., 2015b), such as employment or postcompulsory education, as well as overall quality of life (Lachapelle et al., 2005). Consequently, more research is needed to favor further experiences with the implementation of this program in clinical, educational and community-based settings, thus facilitating appropriate support and facilitating conditions for QoL enhancement (Mumbardó-Adam et al., 2024).

## Electronic supplementary material

Below is the link to the electronic supplementary material.

Supplementary Material 1

Supplementary Material 2

Supplementary Material 3
