# Supplementary material for: Promoting Self-Determination in Young Adults with Autism: A Multicenter, Mixed Methods Study
Source: J Autism Dev Disord. 2025 Feb 15;56(7):2651–70. doi: 10.1007/s10803-025-06739-6 (PMC13346146; doi:10.1007/s10803-025-06739-6)
Supplement: Supplementary file 1 — Supplementary Material 1 [file 10803_2025_6739_MOESM1_ESM.docx]

**Supplementary Material 1**

*Measures to Assess the Characteristics of Participants*

| Measures | Assessment | Administration | Reliability and validity |
| --- | --- | --- | --- |
| Autism Diagnostic Observation Scale – Second Edition  (ADOS-2: Module 4; Lord et al. (2015) | Autism condition | The ADOS-2 is a standardized and semi-structured assessment of communication, social interaction, and the play or imaginative use of materials by individuals suspected of having autism. It consists of five modules corresponding to different ages and language development and takes between 40 and 60 minutes to administer. Module 4 of the ADOS-2 was administered to adolescents and adults with fluent language. | The instrument has reliability and validity evidence with intraclass correlations ranging from .82 to .93, test-retest reliability with correlation coefficients between .74 to .90, and excellent internal consistency, with an alpha value higher than .90. This test was administered by staff accredited in its application and evaluation. |
| Social Communication Questionnaire – Part B  (SCQ-B; Rutter et al., 2019) | Autism condition | This 40-item questionnaire is responded by parents and provides information about social and communication behaviors, as well as observed interests. It consists of two parts: Part A refers to past life, and Part B focuses on behavior over the last three months. Part B was used in this study. | The instrument has a high internal consistency (α = .87 to .93), a good construct validity (with correlations with the ADI-R between .68 to .79), and a strong concurrent validity. |
| Wechsler Adult Intelligence Scale-IV  (WAIS-IV: Verbal Comprehension Index; Wechsler, 2012) | Verbal comprehension | This clinical instrument is designed to assess general intellectual ability in people between 16 and 89 years and 11 months of age. The main verbal comprehension subtests (Similarities, Vocabulary, and Information) were used to assess participants’ verbal comprehension. | The instrument has a high internal consistency in all its subtests, ranging from α = .70 to .95. It also has a good construct validity, with factor loadings above .70 in the four factors. It has a good concurrent validity, with high correlations with the Wechsler Intelligence Scale for Children-IV (from .60 to .80) and predictive validity with correlation coefficients between .50 to .70. |

**Supplementary Material 1 (continuation)**

*Measures to Assess the Characteristics of Participants*

| Measures | Assessment | Administration | Reliability and validity |
| --- | --- | --- | --- |
| Adaptive Behavior Assessment System-II (ABAS-II; Harrison & Oackland, 2003) - The proxy-reported adult version of the Spanish adaptation of Montero and Fernández-Pinto (2013) was used. | Adaptive behavior | This instrument can be self or proxy reported and consists of 239 items that assess the adaptive skills of people with and without disabilities. Informants indicate how often the individual performs each activity with a 4-point scale (Not able, Never when needed, Sometimes when needed, Always when needed). It consists of ten adaptive skill areas: Communication, Community Resource Utilization, Functional Academic Skills, Home Living, Health and Safety, Leisure, Self-Care, Self-Direction, Social, and Work. These areas are also grouped into three domains: Conceptual, Social and Practical. A General Adaptive Behavior Index (GAC) can also be obtained. | Internal consistency is high for the CAG (α=.98) and the three dimensions (α=.95 - .97) and good for the 10 skills (α=.82 - .96). Factor analysis also shows a good internal structure for the adult proxy-report (CFI: .90, TLI: .86, SRMR, .08) (Montero and Fernandez-Pinto, 2013). |
