# Supplementary material for: Promoting Self-Determination in Young Adults with Autism: A Multicenter, Mixed Methods Study
Source: J Autism Dev Disord. 2025 Feb 15;56(7):2651–70. doi: 10.1007/s10803-025-06739-6 (PMC13346146; doi:10.1007/s10803-025-06739-6)
Supplement: Supplementary file 2 — Supplementary Material 2 [file 10803_2025_6739_MOESM2_ESM.docx]

**Supplementary Material 2**

*Questions of the focus groups*

| Construct | Questions |
| --- | --- |
| Effectiveness |  |
| Volitional actions | How do you feel about your ability to do things on your own? |
|  | When we set out to do something, we have seen that sometimes we find it very difficult to do it. How has that changed? What has made it easier or harder for you to do it? |
| Agentic actions | Are you any closer to achieving any of your goals? |
|  | What changes or steps have you made to achieve your goals? |
|  | What did you do when you encountered a problem that made it difficult to achieve your goals? What did you do in those situations? What helped you overcome them? |
| Action-control beliefs | What has been your experience with the changes you have made? |
|  | How do you think your strengths have helped you take steps toward your goals? |
|  | Which of your skills and strengths do you think will help you solve problems in the future? Why? |
| Acceptability | What do you think of the program? What has it given you? |
|  | What do you think about the group format? |
|  | Would you make any suggestions for change? |
