# Supplementary material for: Promoting Self-Determination in Young Adults with Autism: A Multicenter, Mixed Methods Study
Source: J Autism Dev Disord. 2025 Feb 15;56(7):2651–70. doi: 10.1007/s10803-025-06739-6 (PMC13346146; doi:10.1007/s10803-025-06739-6)
Supplement: Supplementary file 3 — Supplementary Material 3 [file 10803_2025_6739_MOESM3_ESM.docx]

**Supplementary Material 3**

*Themes and Subthemes of the Effectiveness, Implementation, and Acceptability of the Program*

| Dimensions | Themes | Subthemes | Quotations |
| --- | --- | --- | --- |
| Effectiveness | Changes in volitional actions | Autonomy | *I’m making my own decisions (P14)* |
|  |  | Self-initiation | *I see that now I can not only make decisions, which was already difficult for me, but also put it into practice. Now it seems to me that this happens in a much more fluid way (P1)* |
|  | Changes in agentic actions | Self-direction | *Before, I preferred that things went badly for me than to talk to others or to do something, or act. But now it has helped me to have courage, to act and to have those things that help me, that are for my benefit (P8)* |
|  |  | Self-regulation | *If I see that I'm getting overwhelmed, I stop for a moment, go watch a series or something and then I come back (P12)* |
|  | Changes in action-control beliefs | Control expectancy | *Asking for advice and help, among all (P5 when asked what helped him to make better decisions)* |
|  |  | Empowerment | *I think I have a better self-esteem (P4)* |
|  |  | Self-realization | *I have my own assimilation process [of things that happened to her] (P16)* |
|  | Facilitators for change | Personal attitudes and interests | *Make an effort [has helped to achieve goals] (P15)* |
|  |  | Environmental supports | *I have had to deal with more situations and have more experience, which helps (P1)* |
|  |  | People of support | *My friends have helped me to understand that I can say things in a not very assertive way (P4)* |
|  | Barriers for change | Barriers to volitional action | *This is precisely my problem from the beginning. Having to do things and not getting into it (P9)* |
|  |  | Barriers to agentic action | *But the rest of the things don't catch my attention. What I like is the artistic stuff. Everything that is not artistic, I hate it (P6)* |
|  |  | Barriers to action-control beliefs | *I have set myself overly ambitious goals (P6)*  *I don’t know how to use my strengths (P16)* |

| Dimensions | Themes | Subthemes | Quotations |
| --- | --- | --- | --- |
| Implementation | Format | Structure | *The initial part to see how the week has gone works very well. It works as a follow-up of the objectives* |
|  |  | Setting | *We do not have time to do the perspectives activity. We will do it next day* |
|  |  | Materials | *It is tiresome to wait for the questionnaires. The problem is always waiting for everyone to finish, participants have very different answering speeds and there is a risk of boredom in the fastest ones* |
|  | Content | Content evaluation | *Very positive evaluation of the whole session* |
|  |  | Content application | *P1 brings a new perspective to P4 and helps to find possible solutions to her problem* |
|  | Challenges | Participants’ challenges | *[Name of participant] would need weekly individual meetings and specialized treatment for PTSD* |
|  |  | Participation and group cohesion challenges | *It is a group that finds it hard to initiate conversations and connect with each other* |
|  | Improvements | - | *It would be good to do more activities in pairs* |
| Acceptability | Usefulness of the program | - | *Personally, perhaps I have seen an improvement in my ability to adapt to things that may surprise me or to contingencies. And above all to the adaptation to such contingencies (P1)* |
|  | Advantages of the group format | - | *It's good to do it in group, because you see what other people think, and they also have problems like you (P10)* |
|  | Suggestions for improvement | - | *Maybe do one week in group format and another week in individual format. Something like that, for example (P11)* |

*Note*. Participants reported efficacy and acceptability. The facilitators of the groups reported implementation.
